# Establishment and characterization of a novel untransfected corneal endothelial cell line from New Zealand white rabbits

**Published:** 2009-05-29

**Authors:** Tingjun Fan, Dansheng Wang, Jun Zhao, Jing Wang, Yongfeng Fu, Ruichao Guo

**Affiliations:** 1 Research Institute of Corneal Tissue Engineering, Ocean University of China, Shandong, China; 2 Department of Marine Biology, College of Marine Life Sciences, Ocean University of China, Shandong, China; 3 School of Agriculture, Eastern Liaoning University, Liaoning, China; 4 Shanghai Medical College, Fudan University, Shanghai, China

## Abstract

**Purpose:**

To establish and characterize a novel untransfected corneal endothelial cell line from New Zealand white rabbits (NRCE cell line) for studies on corneal endothelial cells.

**Methods:**

Primary culture was initiated with a pure population of NRCE cells from corneal endothelia by successive detachment and reattachment procedure of different durations, and cultured in 20% fetal bovine serum-containing DMEM/F12 media with several supplements. The cell line was characterized by chromosome analysis, fluorescence immunoassay and reverse transcription PCR. The tumorigenic potency of the cell line was examined by subcutaneous inoculation to nude mice. The biocompatibility of the cell line to denuded amnions was examined with routine microscopic and electron microscopic techniques.

**Results:**

NRCE cells in primary culture proliferated to confluency in 25 days and has been subcultured to passage 227 to date. The novel NRCE cell line, with a steady growing rate in 20% bovine calf serum (BCS)-containing DMEM/F12 medium and a population doubling time of 40.32 h at passage 191, has been established. NRCE cells exhibited chromosomal aneuploidy but their modal chromosome number was still 44. The results of gene expression patterns of marker proteins and membrane transport proteins, combined with immunofluorescent localization patterns of cell junction proteins, indicated that NRCE cells retained normal corneal endothelial characteristics and normal expression pattern of functional proteins. Furthermore, these cells, without any tumorigenic potency, had excellent biocompatibility to denuded amnions in 20% BCS-containing DMEM/F12 medium, and formed confluent cell sheets attached tightly to denuded amnions.

**Conclusions:**

These results suggest that a novel untransfected NRCE cell line established here maintains normal corneal endothelial characteristics and potencies to form normal cell junctions and perform normal functions of transmembrane transport.

## Introduction

Mammalian corneal endothelium (MCE), essential for maintaining corneal transparency, is composed of a monolayer of hexagonal endothelial cells with limited regenerative capacity after birth [1]. When too much MCE cells are destroyed by disease or trauma, corneal edema and blindness ensue. This kind of ensued blindness, also called primary corneal endotheliopathy, can be cured by corneal transplantation with normal donor corneas [2]. It is a great pity that most of the sufferers cannot be cured by corneal transplantation due to a lack of donor corneas [3].

Since abundant MCE cells are absolutely necessary for studies on corneal endothelial cells and corneal equivalent reconstruction, attempts at establishing normal corneal endothelial cells have been made in rats, rabbits, bovine, cats, and human beings [4-10]. Several immortalized MCE cell lines have been established by transformation of primary endothelial cells with viral oncogenes [8-10]. However, these immortalized cell lines cannot be used for biological study and reconstruction of tissue-engineered corneas because of their abnormal phenotypes and latent risk of tumorigenicity [10]. Till now, primary cultures of MCE cells have been initiated successfully, but no untransfected MCE cell lines have been established except from the domestic rabbit *Oryctolagus curiculus* [4-7].

New Zealand white rabbits, a kind of widely used experimental mammals, play vital roles in medical sciences such as toxicity testing, pyrogen testing, drug development, and corneal transplantation. Recently, they become the most frequently used mammalian models for the evaluation of tissue-engineered cornea transplantation [11,12]. Unfortunately, no untransfected corneal endothelial cell line from New Zealand white rabbits (NRCE cell line) had been established by now. This study was intended to establish a novel untransfected NRCE cell line and characterize its inherent property, protein expression pattern, tumorigenic potency, and its biocompatibility to denuded amnions.

## Methods

### Materials

Amnions were obtained from The Eye Research Institute of Shandong Medical Academy and their epithelia were denuded by 0.25% trypsin (Sigma-Aldrich, St. Louis, MO). Rabbit corneal keratocytes, from a rabbit corneal keratocyte cell line established in our laboratory, were cultured in 20% bovine calf serum (BCS)-containing Dulbecco’s Modified Eagle’s Medium/Ham’s Nutrient Mixture F12 (DMEM/F12, 1:1) medium (Invitrogen, Carlsbad, CA) at 37 ^°^C with 5% CO_2_.

### Animals

Four female New Zealand white rabbits (age range, 4–5 weeks) were used and all animal protocols were approved by the Experimental Animal Center of Shandong Medical Academy, in accordance with the ARVO Statement for the Use of Animals in Ophthalmic and Vision Research.

### In vitro culture of NRCE cells

Fresh eyeballs, obtained from gas-ether anaesthetized New Zealand white rabbits, were washed twice with mercuric chloride (1:5,000) and disinfected in 1% gentamycin for 20 min. Each cornea was cut off and 0.25% trypsin was infused into the hollow of flatly placed corneas. Two minutes later, trypsin solution was discarded and the corneas were rinsed with 10% BCS-containing DMEM/F12 medium (Invitrogen). The corneas were quartered and attached into the gelatin-coated wells of a 24 well tissue culture plate with their endothelial side down, and cultured in 10% fetal bovine serum (FBS, Invitrogen)-containing DMEM/F12 medium at 37 ^°^C with 5% CO_2_. The corneal fragments were detached and reattached sequentially into a new well for different times from 6 to 48 h. Cells in the wells containing only a pure population of NRCE cells were collected and re-suspended in 20% FBS-containing DMEM/F12 medium with antibiotics, and plated into two new wells. The culture medium was further supplemented with basic fibroblast growth factor (bFGF, 10 ng/ml), epidermal growth factor (EGF, 10 ng/ml), N-acetylglucosamine hydrochloride (50 μg/ml), glucosamine hydrochloride (50 μg/ml), chondroitin sulfate (0.8 mg/ml), oxidation-degradation products of chondroitin sulfate (50 μg/ml; all from Sigma-Aldrich), carboxymethyl-chitosan (50 μg/ml; AK Scientific, Mountain, CA), bovine ocular extracts (5 ng/ml; Bosen Biological Pharmaceutical Inc., Xi’an, China) and culture supernatant of rabbit corneal keratocytes at logarithmic phase (0.3 ml in 5 ml medium), and was refreshed at 5 day intervals during primary culture. After a monolayer was established, NRCE cells were subcultured with 0.25% trypsin as described previously [7]. From passage 55, NRCE cells were subcultured in 20% BCS-containing DMEM/F12 medium but without growth factors and the other supplements as described above.

### Growth properties of the NRCE cell line

Characterization of the growth properties of passage 191 NRCE cells were performed according to the procedure described by Fan et al. [7]. The density of NRCE cell suspension used for culture initiation was 1.73×10^5^ cell/ml, and the cells were cultured in 20% BCS-containing DMEM/F12. Three independent measurements were performed and the mean value of cell density was calculated.

### Chromosome analysis

Chromosome analysis of passage 191 NRCE cells, cultured in 20% BCS-containing DMEM/F12 medium, was also performed as described previously by Fan et al [7]. The cells at logarithmic phase were administered with 60 μg/ml of colchicine (Sigma-Aldrich) for 10 h at 37 ^°^C.

### Determination of protein expression by immunocytochemistry

For immunofluorescence studies, passage 191 NRCE cells were plated into a 24 well culture plate and cultured in 20% BCS-containing DMEM/F12 medium as described above. Once 90% confluency was reached, the cells were washed 3 times with phosphate-buffered saline (PBS). Then the cells were treated with 0.5% Triton X-100 (Sigma-Aldrich) for 20 min at 37 ^°^C after fixed with 4% paraformaldehyde (Sigma-Aldrich) for 15 min. After blocking with 5% BCS in PBS for 30 min, the cells were washed with PBS and then incubated, respectively, with goat anti-human zonula occludens protein 1 polyclonal antibody (1:50; Santa Cruz Biotechnology, Santa Cruz, CA), mouse anti-human N-cadherin monoclonal antibody (1:50; Santa Cruz Biotechnology), mouse anti-human connexin 43 monoclonal antibody (1:250; Chemicon, Temecula, CA) and mouse anti-human integrin αv/β5 monoclonal antibody (1:50; Santa Cruz Biotechnology) at 37 ^°^C for 90 min. The cells were washed four times with PBS and then incubated with fluorescein (FITC)-conjugated rabbit anti-goat IgG antibody (1:100) or FITC-conjugated goat anti-mouse IgG antibody (Biosynthesis Biotechnology, Beijing, China) at 37 ^°^C for 90 min. The cells were washed five times with PBS and visualized with a Nikon Eclipse TE2000-U inverted microscope. Omission of primary antibodies was used as negative controls.

### Determination of gene expression by reverse transcription PCR

For determination of gene expression of marker proteins, primary cultured cells and established cell lines at passage 191 were cultured in 20% BCS-containing DMEM/F12 medium as described above. Total RNA was extracted with NucleoSpin RNAII Assay Kit (Macherey-Nagel, Germany). The first strand cDNA was synthesized from 2 μg of the total RNA in a 20 μl reaction mixture with PrimeScript^TM^ 1st strand cDNA Synthesis Kit (Takara, Ishiyama, Japan). Target genes of collagen type IV α2 (*COL4A2*), *keratin 12*, *FLK1* (vascular endothelial growth factor receptor 2) and Von Willebrand factor (*vWF*; Table 1) were then amplified in a 20 μl volume containing Tag PCR MasterMix (Tiangen Biotech, Bejing, China) for 37 cycles by touchdown PCR using the following parameters: 5 min at 94 °C, 10 cycles of step-down PCR consisting of 1 min at 94 °C, 50 s at 57 °C then decrease 0.5 °C each cycle until 52 °C; 1 min at 72 °C, followed by 27 cycles of 1 min at 94 °C, 50 s at 52 °C, 1 min at 72 °C, with a final extension of 5 min at 72 °C. PCR products were run on a preparative 2% agarose gel, and the bands visualized under UV illumination. *β-actin* was used as a loading control.

**Table 1:** Reverse transcription PCR primer sequences.

**mRNA**	**Forward primer (5′−3′)**	**Reverse Primer (5′−3′)**	**Amplicon size**	**Accession number**
*COL4A2*	GTTCCAGGATGTGATGGACA	TCTCCAGTGTCACCTTTGTG	371	L01477
*Keratin12*	GATGCTAATGCTGAGCTCGA	ACCTGCCCTACAGCTTTGTA	393	X77665
*FLK1*	ACGGAACATCCTCTTGTCGG	GCGCTCGCTTGTAACAGGTT	410	AB017155
*vWF*	TTCATGCACTGCACCTCAAGCG	GGCGTCGCACATGGGCT	380	S64544
*VDAC2*	GCAGTGGTGTGGAATTCTCA	TAGTGTGTAGCTGGAAGTCC	441	AF209726
*VDAC3*	ACAGGAAAAGCGTCAGGCAA	TCAAAAGCCAACACAGCCCA	290	AF209727
*AQP1*	TGCCACAGCCAGTGTAGTCG	ACCTCGTCCCTGACCCTGAA	215	AF495880
*ATP1A1*	CTCTGTAACAGGGCGGTATT	ATTGGCGTTGAGGTTCTTAT	219	AF235024
*β-actin*	ATCGTGATGGACTCCGGCGA	AGGAAGGAGGGCTGGAACAG	350	AF309819

Primers were designed to amplify human sequences for collagen type IV alpha 2 chain (*COL4A2*), vascular endothelial growth factor receptor 2 (*FLK1*), von Willebrand factor (*vWF*), voltage-dependent anion channel 2 (*VDAC2*), voltage-dependent anion channel 3 (*VDAC3*), Aquaporin 1 (*AQP1*), and Na^+^-K^+^ ATPase alpha 1 subunit (*ATP1A1*).

For determination of gene expression of membrane transport proteins, target genes of voltage-dependent anion channel 2 (*VDAC2*), voltage-dependent anion channel 3 (*VDAC3*), aquaporin 1 (*AQP1*), and Na^+^/K^+^ ATPase alpha 1 subunit (*ATP1A1*; Table 1), were amplified using the following parameters: 5 min at 94 °C, 10 cycles of step-down PCR consisting of 1 min at 94 °C, 50 s at 55 °C then decrease 0.5 °C each cycle until 50 °C; 1 min at 72 °C, followed by 27 cycles of 1 min at 94 °C, 50 s at 50 °C, 1 min at 72 °C, with a final extension of 5 min at 72 °C. *β-actin* was also used as a loading control.

### Assay of tumorigenic potential of NRCE cell line

Passage 191 NRCE cells at logarithmic phase were collected by trypsin digestion and suspended with serum-free DMEM/F12 medium. Then 0.2 ml of cell suspension with a density of 4.6×10^6^ cell/ml was inoculated subcutaneously into one of the forehand oxters of 4 BalB/c nude mice and the tumorigenic status of the mice were monitored daily. After 45 days, the skin of the oxter of inoculated mice was surgically opened and tumorigenic status was examined. HeLa cells with a density of 4.6×10^6^ cell/ml inoculated into 4 BalB/c nude mice as above were used as positive controls.

### Evaluation of biocompatibility of NRCE cells with denuded amnions

Passage 191 NRCE cells at logarithmic phase were collected and suspended with 20% BCS-containing DMEM/F12 medium. Then 1 ml of NRCE cell suspension with a density of 1.73×10^5^ cell/ml was plated into 24 well culture plates coated with denuded amnions, and cultured at the same conditions as described above. Culture medium was refreshed at 5 day intervals and the morphology and growth status were monitored daily. The morphology of cell sheets formed was examined with microscopic and scanning electron microscopic techniques, and the attachment status of cell sheets and scaffold carriers was examined with transmission electron microscopic techniques.

## Results

### In vitro culture of NRCE cells

**Figure 1:**
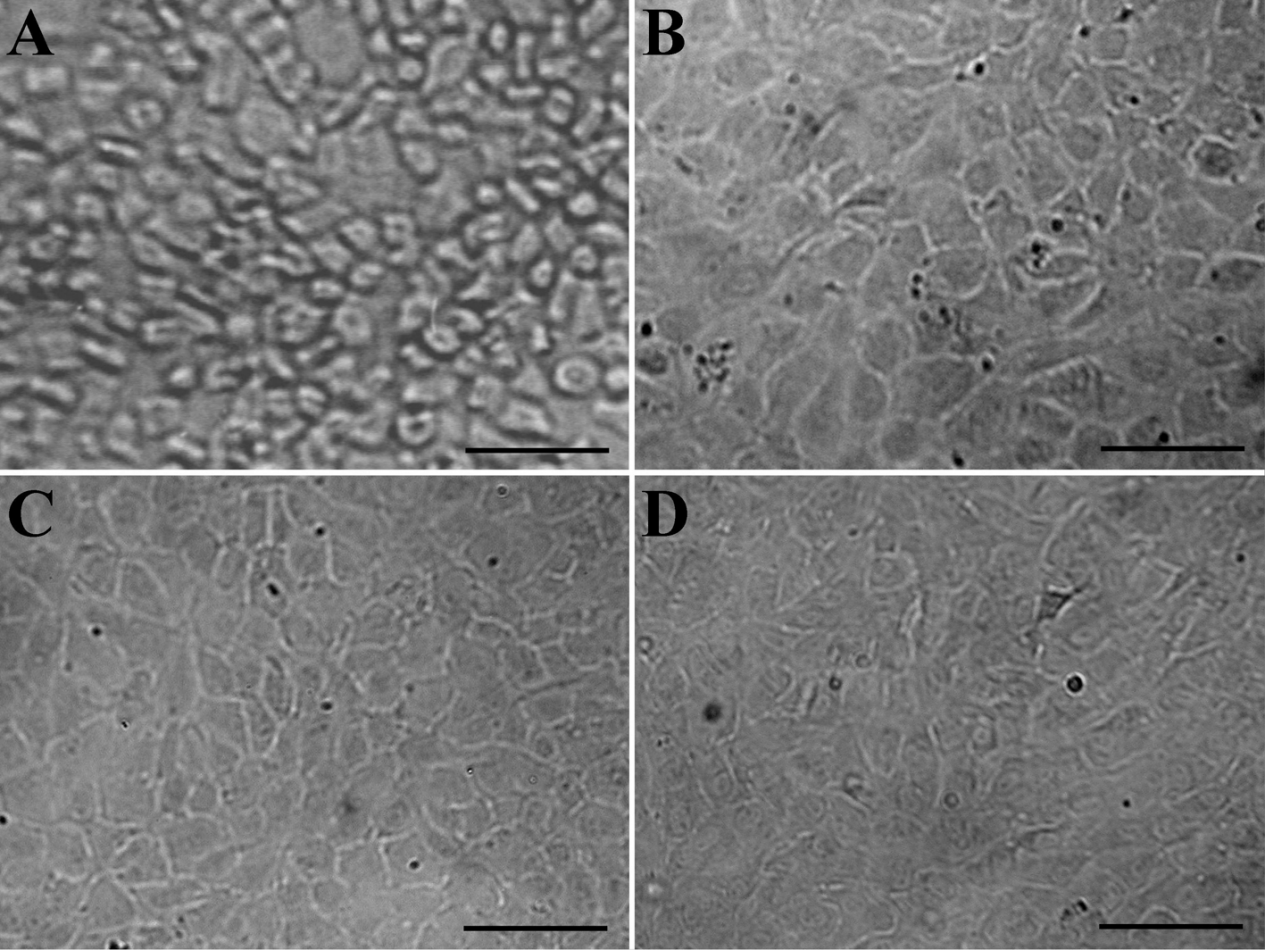
Morphology of NRCE cells in vitro. **A**: Freshly attached NRCE cells from corneal fragments. The non-spread polygonal cell morphology, i.e. corneal endothelial-like, was shown. **B**: The monolayer formed by NRCE cells in primary culture. The plump polygonal cell morphology was shown. **C**: Passage 56 NRCE cells. The elongated polygonal cell morphology was shown. **D**: Passage 227 NRCE cells. The elongated polygonal cell morphology was shown. Scale bar: 100 μm.

**Figure 2:**
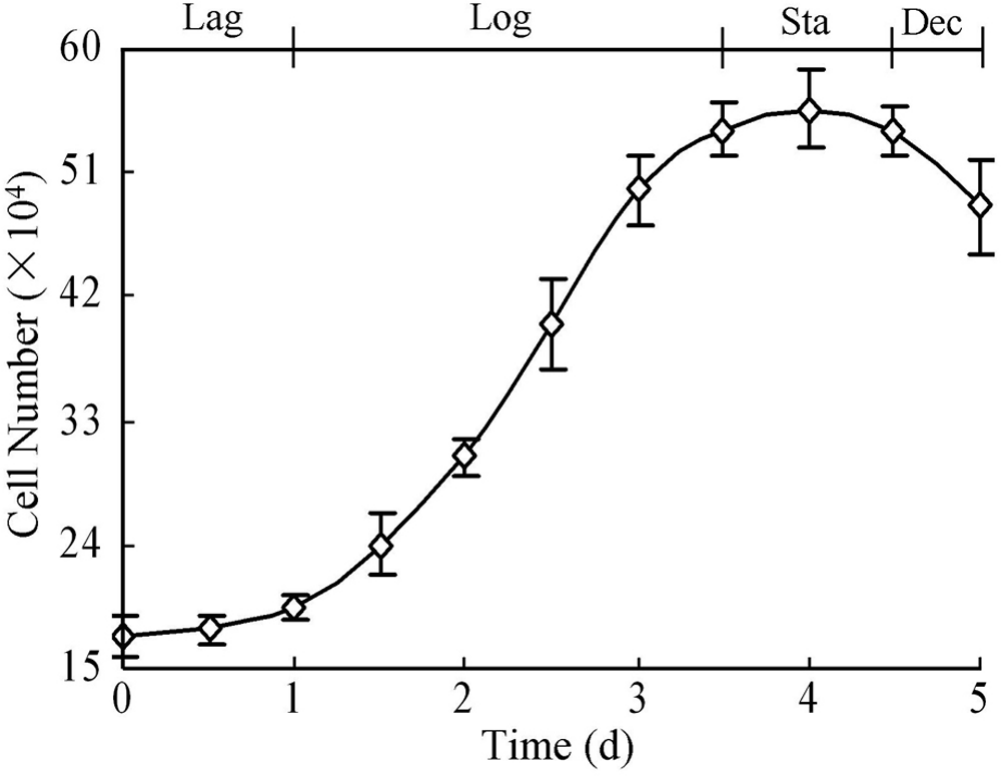
The growth curve of NRCE cells at passage 191. The lag phase (Lag), logarithmic phase (Log), stationary phase (Sta), and decline phase (Dec) were shown.

There were numerous pure NRCE cells attached to the bottom of the wells after the corneal fragments were detached (Figure 1A). These cells were in a typical polygonal morphology and many of them were still in the non-extended state. About 25 days later, the primary cultured NRCE cells grew into a confluent monolayer (Figure 1B). Most of the NRCE cells were plump and in polygonal cell morphology. During subsequent subculture, the polygonal morphology of NRCE cells began to elongate to some extent after the culture medium was replaced with 20% BCS-containing DMEM/F12 medium but no growth factors (Figure 1C). The NRCE cells grew and proliferated at a steady rate, and their doubling time was calculated to be 40.32 h at passage 191 (Figure 2). At present, the NRCE cells have been subcultured to passage 227 (Figure 1D), and a novel continuous untransfected NRCE cell line has been established.

### Chromosome analysis of NRCE cell line

**Figure 3:**
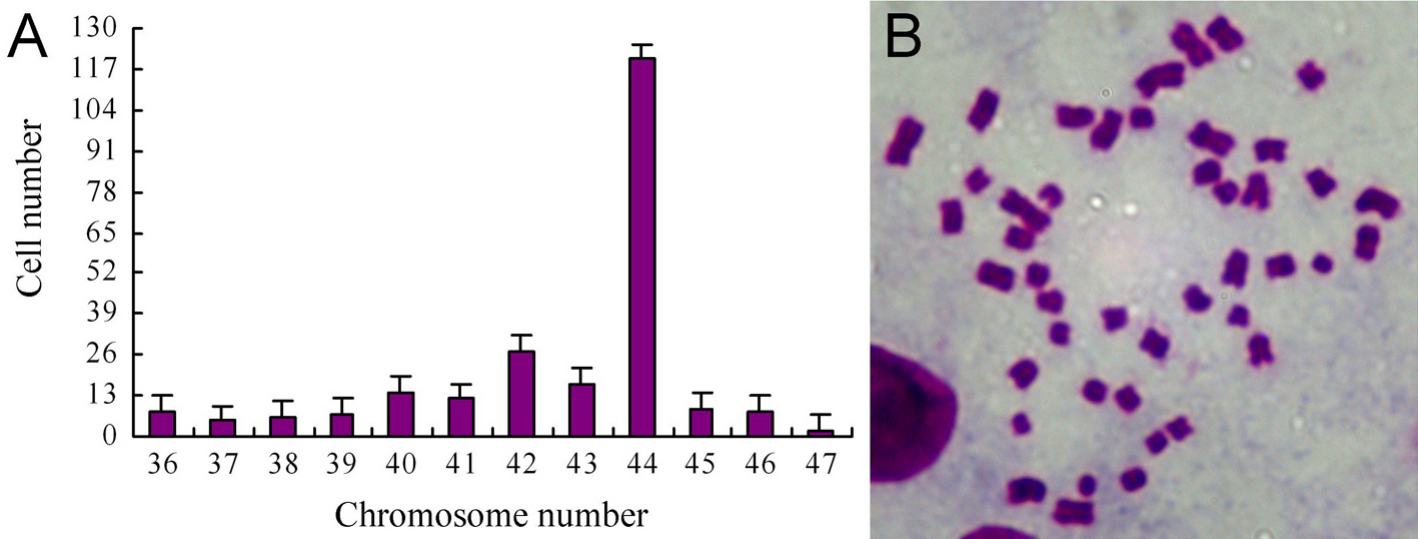
Chromosome analyses of NRCE cells at passage 191. **A**: The chromosomal aneuploidy of NRCE cells with chromosome numbers ranged from 36 to 47. Among them, NRCE cells with chromosome number of 44 were about 51.06%. **B**: Chromosomes from a NRCE cell with a number of 44.

Chromosome analysis showed that NRCE cells at passage 191 exhibited chromosomal aneuploidy with chromosome numbers ranged from 36 to 47 (Figure 3A), but the modal chromosome number of the established NRCE cell line was still 44 (Figure 3B).

### Gene expression of marker proteins and membrane transport proteins

**Figure 4:**
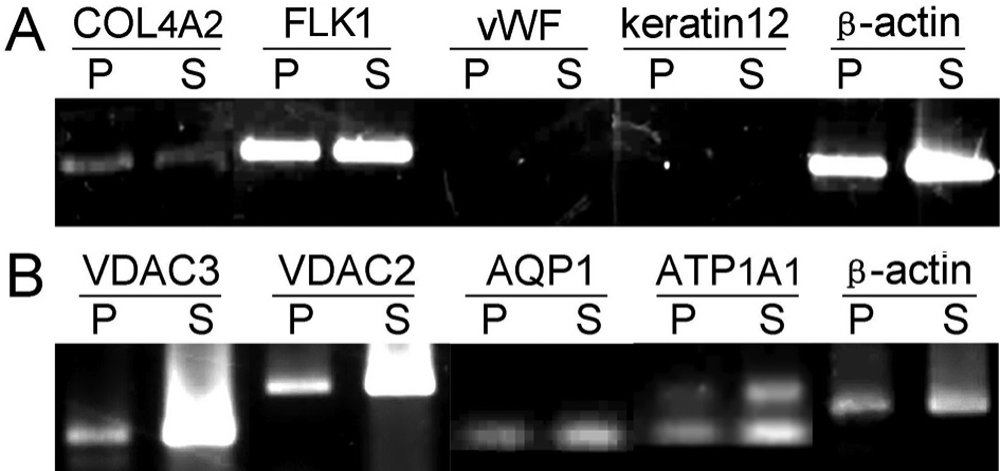
Gene expression of marker proteins and membrane transport proteins in NRCE cells. **A**: Gene expression of marker proteins. NRCE cells stably express collagen type IV α2 chain (*COL4A2*) and vascular endothelial growth factor receptor 2 (*FLK1*), but not von Willebrand factor (*vWF*) or *keratin 12*. **B**: Gene expression of membrane transport proteins. NRCE cells express voltage-dependent anion channel 3 (*VDAC3*), voltage-dependent anion channel 2 (*VDAC2*), aquaporin 1 (*AQP1*), and Na^+^-K^+^ ATPase alpha 1 subunit (*ATP1A1*). P, NRCE cells in primary culture; S, NRCE cells at passage 191. *β-actin* was used as a loading control.

Among the genes of marker proteins detected by reverse transcription PCR, NRCE cells maintained stable expression of *COL4A2* and *FLK1*, but not *vWF* and *keratin 12* (Figure 4A), suggesting that the NRCE cell line is of corneal endothelial origin, not vascular endothelial or corneal epithelial origin. Among the genes of membrane transport proteins detected, NRCE cells maintained stable and strong expression of *VDAC3*, *VDAC2*, *AQP1*, and ATP1A1 (Figure 4B), suggesting that the NRCE cell line still maintains the normal expression pattern of rabbit corneal endothelial cells.

### Expression of cell junction-related proteins

**Figure 5:**
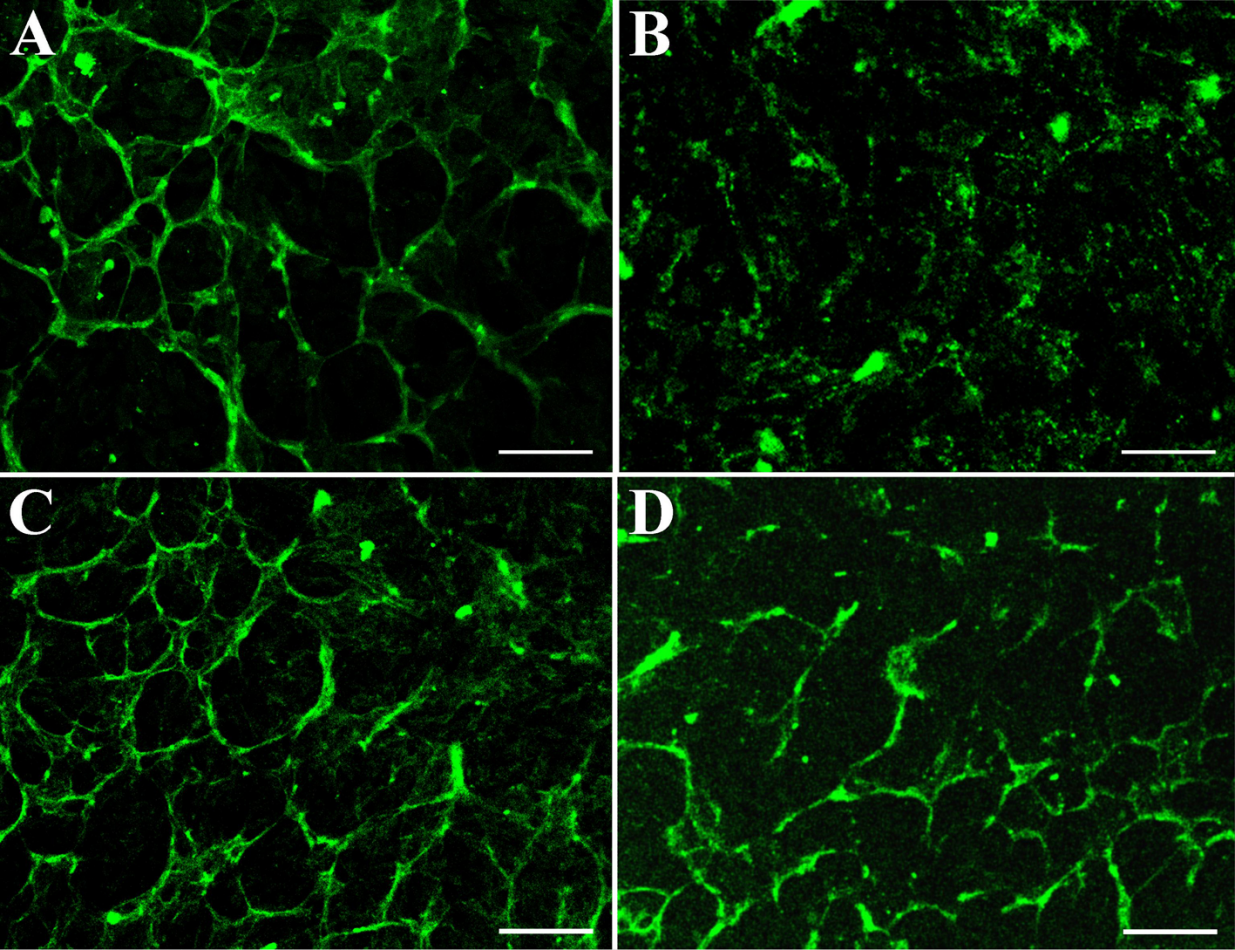
Immunofluorescence staining patterns of junction-related proteins of NRCE cells at passage 191. **A**: zonula occludens protein 1. **B**: E-cadherin. **C**: connexin 43. **D**: integrin αv/β5. Scale bar: 100 μm.

**Figure 6:**
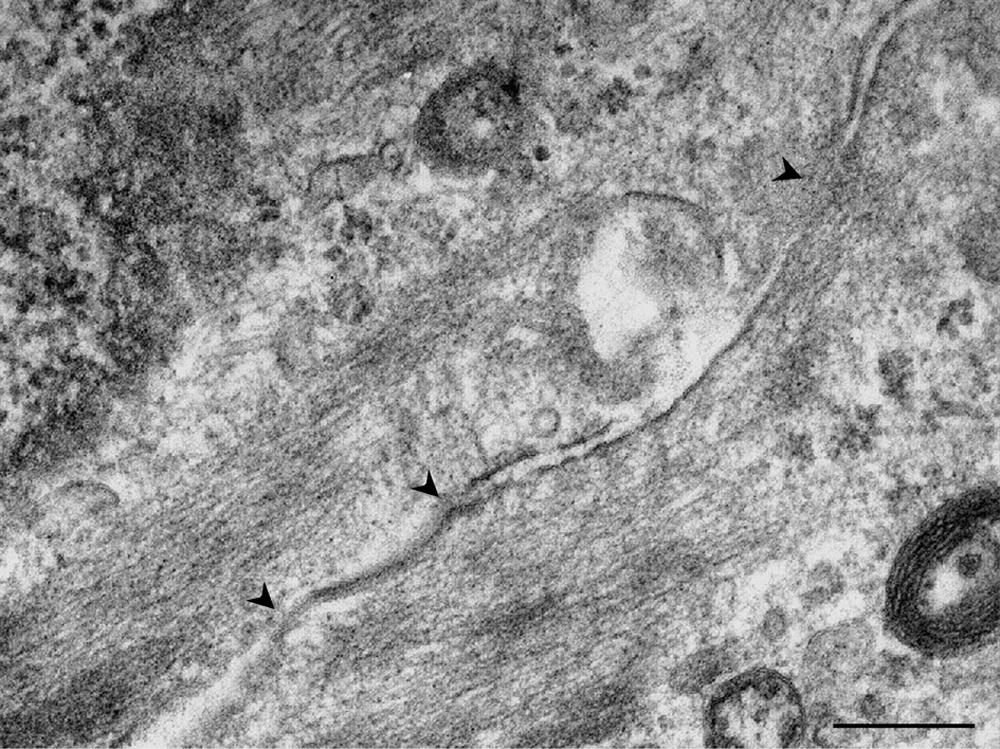
Transmission electron microscopy of NRCE cells at passage 191. Intercellular junctions (arrow heads) were shown. Scale bar: 0.2 μm.

Immunofluorescence staining of cell junction-related proteins showed that NRCE cells maintained stable expression of zonula occludens protein 1, E-cadherin, connexin 43, and integrin αv/β5 (Figure 5). Various cell junctions were found between NRCE cells under transmission electron microscope (Figure 6). These results suggested that the established NRCE cell line still maintains the normal expression pattern of cell junction-related proteins of rabbit corneal endothelial cells.

### Assay of tumorigenic potential of NRCE cells

Solid tumors were found in all 4 nude mice 8 days after inoculated with HeLa cells. However, no solid tumor was found in 4 BalB/c nude mice 45 days after passage 191 NRCE cells were inoculated. The results implied that the established NRCE cell line has no tumorigenic potency.

### Biocompatibility of NRCE cells with denuded amnions

**Figure 7:**
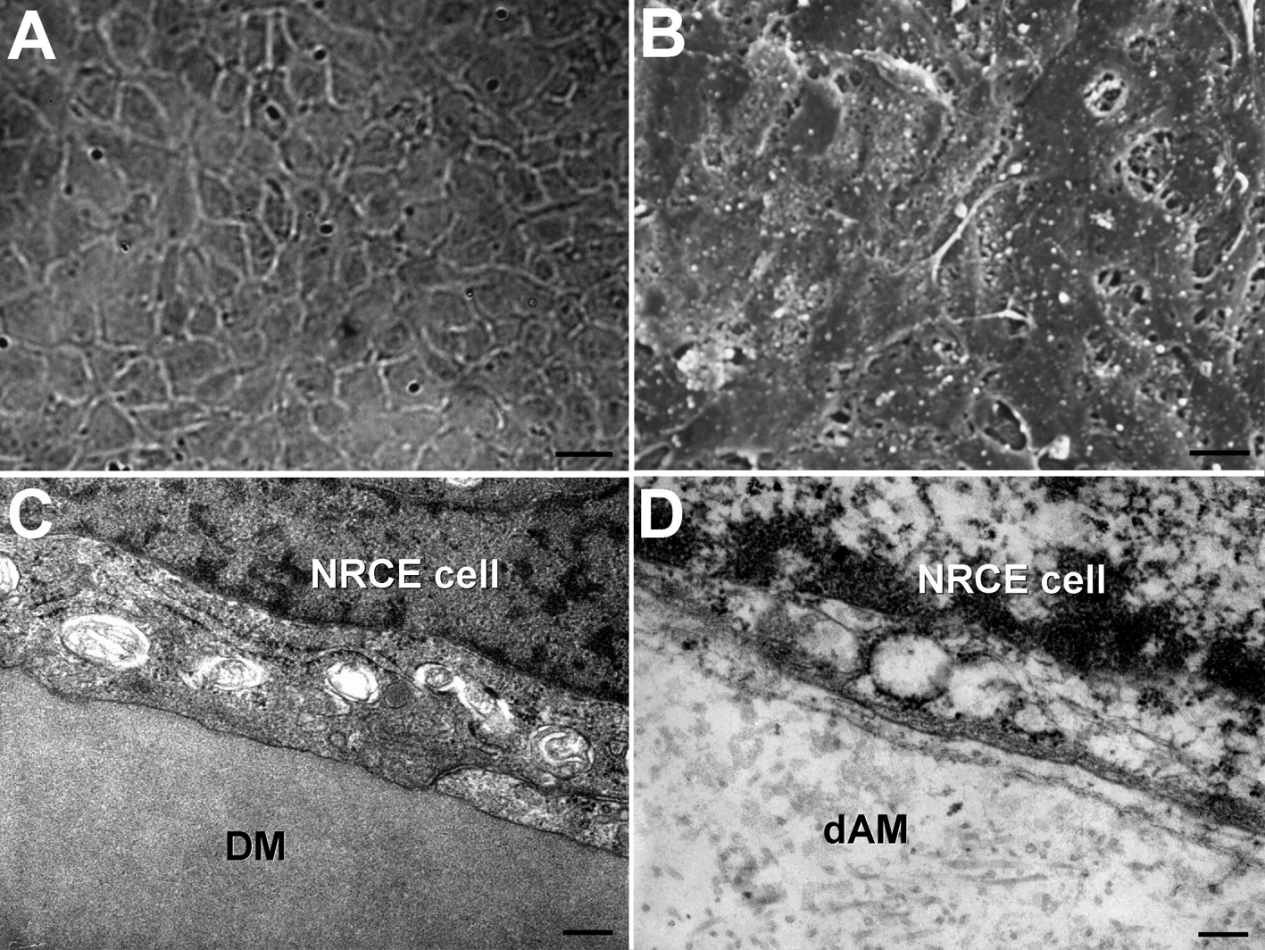
Evaluation of biocompatibility of the passage 191 NRCE cells on denuded amnion. **A**: NRCE cells grew into a confluent cell sheet on denuded amnion 90 h later. Scale bar: 40μm. **B**: SEM image showing the cell morphology of a NRCE cell sheet on denuded amnion. Scale bar: 10 μm. **C**: TEM image of a NRCE cell from a live rabbit corneal endothelium on Descement's membrane (DM). Scale bar: 0.2 μm. **D**: TEM image of a NRCE cell on denuded amnion. The tight attachment status of the NRCE cell to denuded amnion (dAM), almost identical to that of the NRCE cell on Descement's membrane in (**C**), was shown. Scale bar: 0.2 μm.

Passage 191 NRCE cells grew very well on denuded amnions, and confluent cell sheets formed 90 h later (Figure 7A,B). In addition, the cell sheets established tight attachments to denuded amnions, almost identical to that between NRCE cells and Descement's membranes in vivo (Figure 7C,D). These results indicated that the established NRCE cell line has excellent biocompatibility with denuded amnions.

## Discussion

MCE cell lines are necessary in studies of corneal endothelial cells and reconstruction of the ocular surface with a tissue-engineered cornea [7-10]. Even though numerous attempts have been made to establish MCE cell lines, difficulties in obtaining sufficiently pure corneal endothelial cells and inducing their proliferation need to be overcome before initiation of primary culture and successful subculture [5-10].

To obtain sufficiently pure corneal endothelial cells, different efforts were made to initiate the in vitro culture of MCE cells, such as scraping corneal buttons to gather endothelial cells, enzymatic removal of endothelial cells from cornea, and direct corneal fragment attachment [6,7,13,14]. To obtain pure enough NRCE cells for primary culture, strictly controlled trypsin digestion, direct corneal fragment attaching, and successive detaching-reattaching of different durations, have been utilized in this study. These manipulations are key premises for successful initiation of primary culture in this study.

To induce corneal endothelial cell proliferation, attempts have been made to replenish the culture medium with different supplements [15]. Among them, chondroitin sulfate, a component of extracellular matrix (ECM) in the corneal stromal layer, has been frequently used in primary culture of corneal endothelial cells and has been shown to have a positive effect on cell attachment, growth, and proliferation at high concentrations (0.8-25 mg/ml) [14,16]. Glucosamine hydrochloride (the main component of chondroitin sulfate) and N-acetylglucosamine hydrochloride (an acetyl form of glucosamine hydrochloride) were also found to have a positive effect on cell attachment and growth [17,18]. Growth factors such as EGF and bFGF have also been found to have a positive inducing effect on the proliferation of mammalian corneal endothelial cells [14,19-22]. Researchers also found that ocular extracts, containing different factors beneficial for eye metabolism and wound healing, could stimulate the proliferation of corneal endothelial cells [23]. Culture supernatant of corneal keratocytes at logarithmic phase contained plenty of secreted materials including ECM elements (such as collagen and proteoglycans), cytokines, and growth factors that had been shown to stimulate cell attachment, survival, and proliferation [7,24]. Addition of all these supplements in the culture medium is most probably the key premise of inducing cell proliferation and successful subculture of NRCE cells in this study.

With relatively pure NRCE cells and supplement-induced proliferation, a novel untransfected NRCE cell line has been established successfully in this study. The cell line, with the modal chromosome number of 44 and a population doubling time of 40.32 h at passage 191, has been subcultured to passage 227 to date.

Expression patterns of marker proteins are frequently utilized in MCE cell line characterization [10,25-27]. Collagen type IV is one major type of collagen in corneal Descemet's membrane secreted from corneal endothelial cells that can be used as a marker protein of MCE cells [10,26]. FLK1, VEGF receptor 2, is a marker protein of both vascular and corneal endothelial cells [27]. vWF, a large multimeric blood glycoprotein required for normal hemostasis, is a marker protein of vascular endothelial cells [28]. Keratin-12, an intermediate filament protein in basal and suprabasal corneal epithelial cells, is a marker protein of MCE cells [10,29]. In this study, the expression of collagen type IV a2 and FLK1, but not vWF and keratin 12, suggests that the established NRCE cell line is of a corneal endothelial origin, not of vascular endothelial or corneal epithelial origin, which is consistent with the results obtained in human corneal endothelial cells [30] and rabbit corneal endothelial cells [7,10].

Membrane transport proteins of MCE cells, such as VDACs, aquaporin 1, and Na^+^-K^+^ ATPase, play crucial roles in maintaining corneal dehydration and transparency [31,32]. Among these, VDACs are essential for anion transport [33,34], aquaporin 1 (AQP1) for osmotically driven water transport [35], and Na^+^-K^+^ ATPase for maintaining corneal dehydration and transparency [36]. In this study, the maintenance of normal gene expression of VDAC2, VDAC3, aquaporin 1, and Na^+^-K^+^ ATPase indicated that the established NRCE cell line still has potencies to carry out normal functions of transmembrane transport, which coincides with the results obtained from immortalized NRCE cell lines [10].

Since cell junctions are absolutely necessary for MCE cells to maintain the intact endothelium for achieving stable corneal hydration status [37], verification of the expression of junction proteins of NRCE cells was of great importance in cell line characterization. So far, MCE cells are found to have tight junction-associated proteins [38], gap junction-associated proteins [39], anchoring junction-associated proteins [40], and these adhesion junction-associated proteins mediate and strengthen close cell-cell and cell-matrix associations [18,41]. The results of fluorescent immunocytochemistry in this study showed that NRCE cells maintained stable expression of zonula occludens protein 1 (an intercellular tight junction-associated protein), connexin-43 (an intercellular gap junction-associated proteins), N-cadherin (an intercellular anchoring junction-associated protein), and integrin αv/β5 (a cell-matrix anchoring junction-associated protein), and the expression levels of these proteins were similar to those of NRCE cells in vivo (data not shown). All these results suggested that the established NRCE cell line still maintains potency to establish normal cell-cell and cell-extracellular matrix junctions. This expression pattern of cell junction proteins in NRCE cells is different from that of in vitro cultured human corneal endothelial cells [42].

Established cell lines, especially those immortalized by oncogene transfections, may have latent potency of tumorigenicity and abnormal phenotypes [7,10]. Nude mice were utilized to verify the tumorigenic potency of the established cell line in this study. It was found that the established NRCE cell line had no latent potency for tumorigenicity, implying that the NRCE cell line can be used safely for studies of normal corneal endothelial cells and reconstruction studies of tissue-engineered corneas in rabbit models.

Good biocompatibility between corneal cells and scaffold carriers is a vital precondition for reconstruction of tissue-engineered corneas. In this study, NRCE cells, cultured in a growth factor-free medium, could form confluent cell sheets on denuded amnions. The cell sheets were tightly attached to denuded amnions, almost identical to that of rabbit corneal endothelium in vivo. The excellent biocompatibility with denuded amnions implies that the established cell line might be feasible for reconstruction studies of tissue-engineered corneal endothelia [12,43].

In conclusion, a novel continuous untransfected NRCE cell line with normal characteristics and functional protein expression has been established from New Zealand white rabbits, and this cell line can be used for studies of normal corneal endothelial cells and reconstruction of tissue-engineered corneal endothelia in rabbit models. Functional studies of NRCE cells and reconstruction of tissue-engineered rabbit corneal endothelia are underway in our laboratory.

## References

[1] Davies PD, Kirkham JB, Villanueva S (1976). Surface ultrastructure of human donor corneal endothelium.. Trans Ophthalmol Soc U K.

[2] Waring GO, Bourne WM, Edelhauser HF, Kenyon KR (1982). The corneal endothelium. Normal and pathological structure and function.. Ophthalmology.

[3] Wei LN, Zu LH, Gao YQ, Zhang S (1993). Epidemiological investigation of corneal blindness and low vision in China.. J Pract Ophthalmol.

[4] Mimura T, Amano S, Usui T, Araie M, Ono K, Akihiro H, Yokoo S, Yamagami S (2004). Transplantation of corneas reconstructed with cultured adult human corneal endothelial cells in nude rats.. Exp Eye Res.

[5] Baum JL, Niedra R, Davis C, Yue BY (1979). Mass culture of human corneal endothelial cells.. Arch Ophthalmol.

[6] Savion N, Isaacs JD, Shuman MA, Gospodarowicz D (1982). Proliferation and differentiation of bovine corneal endothelial cells in culture.. Metab Pediatr Syst Ophthalmol.

[7] Fan T, Zhao J, Fu Y, Cong R, Guo R, Liu W, Han B, Yu Q, Wang J (2007). Establishment of a novel corneal endothelial cell line from domestic rabbit, *Oryctolagus curiculus.*. Sci China C Life Sci.

[8] Wilson SE, Lloyd SA, He YG, McCash CS (1993). Extended life of human corneal endothelial cells transfected with the SV40 large T antigen.. Invest Ophthalmol Vis Sci.

[9] Joo CK, Pepose JS, Fleming TP (1994). *In vitro* propagation of primary and extended life span murine corneal endothelial cells.. Invest Ophthalmol Vis Sci.

[10] Shin JS, Jang IK, Kim CW, Kim JC (2004). Development and characterization of a rabbit corneal endothelial cell line.. Jpn J Ophthalmol.

[11] Lai JY, Chen KH, Hsiue GH (2007). Tissue-engineered human corneal endothelial cell sheet trans-plantation in a rabbit model using functional biomaterials.. Transplantation.

[12] Hitani K, Yokoo S, Honda N, Usui T, Yamagami S, Amano S (2008). Transplantation of a sheet of human corneal endothelial cell in a rabbit model.. Mol Vis.

[13] Pistsov MY, Sadovnikova EY, Danilov SM (1988). Human corneal endothelial cells: isolation, characterization and long-term cultivation.. Exp Eye Res.

[14] Engelmann K, Bohnke M, Friedl P (1988). Isolation and long-term cultivation of human corneal endothelial cells.. Invest Ophthalmol Vis Sci.

[15] Engelmann K, Bednarz J, Böhnke M (1999). Endothelial cell transplantation and growth behavior of the human corneal endothelium.. Ophthalmologe.

[16] Hsieh P, Baum J (1985). Effects of fibroblastic and endothelial extracellular matrices on corneal endothelial cells.. Invest Ophthalmol Vis Sci.

[17] Iida J, Meijne AM, Oegema TR, Yednock TA, Kovach NL, Furcht LT, McCarthy JB (1998). A role of chondroitin sulfate glycosaminoglycan binding site in alpha4beta1 integrin-mediated melanoma cell adhesion.. J Biol Chem.

[18] Praus R, Brettschneider I (1970). Presence of a non-sulphated glucosaminoglycan in embryonic cornea.. FEBS Lett.

[19] Samples JR, Binder PS, Nayak SK (1991). Propagation of human corneal endothelium *in vitro* effect of growth factors.. Exp Eye Res.

[20] Schultz G, Cipolla L, Whitehouse A, Eiferman R, Woost P, Jumblatt M (1992). Growth factors and corneal endothelial cells: III. Stimulation of adult human corneal endothelial cell mitosis *in vitro* by defined mitogenic agents.. Cornea.

[21] Hoppenreijs VP, Pels E, Vrensen GF, Oosting J, Treffers WF (1992). Effects of human epidermal growth factor on endothelial wound healing of human corneas.. Invest Ophthalmol Vis Sci.

[22] Rieck P, Oliver L, Engelmann K, Fuhrmann G, Hartmann C, Courtois Y (1995). The role of exogenous/endogenous basic fibroblast growth factor (FGF2) and transforming growth factor beta (TGF beta-1) on human corneal endothelial cells proliferation *in vitro.*. Exp Cell Res.

[23] Barritault D, Arruti C, Courtois Y (1981). Is there a ubiquitous growth factor in the eye? Proliferation induced in different cell types by eye-derived growth factors.. Differentiation.

[24] Ren R, Hutcheon AE, Guo XQ, Saeidi N, Melotti SA, Ruberti JW, Zieske JD, Trinkaus-Randall V (2008). Human primary corneal fibroblasts synthesize and deposit proteoglycans in long-term 3-D cultures.. Dev Dyn.

[25] Böhnke M, Vogelberg K, Engelmann K (1998). Detection of neurone-specific enolase in long-term cultures of human corneal endothelium.. Graefes Arch Clin Exp Ophthalmol.

[26] Ljubimov AV, Burgeson RE, Butkowski RJ, Michael AF, Sun TT, Kenney MC (1995). Human corneal basement membrane heterogeneity: topographical differences in the expression of type IV collagen and laminin isoforms.. Lab Invest.

[27] Yang X, Cepko CL (1996). Flk-1, a receptor for vascular endothelial growth factor (VEGF), is expressed by retinal progenitor cells.. J Neurosci.

[28] Sadler JE (1998). Biochemistry and genetics of von Willebrand factor.. Annu Rev Biochem.

[29] Liu CY, Zhu G, Westerhausen-Larson A, Converse R, Kao CW, Sun TT, Kao WW (1993). Cornea-specific expression of K12 keratin during mouse development.. Curr Eye Res.

[30] Engelmann K, Friedl P (1995). Growth of human corneal endothelial cells in a serum-reduced medium.. Cornea.

[31] Riley MV, Winkler BS, Czajkowski CA, Peters MI (1995). The roles of bicarbonate and CO_2_ in transendothelial fluid movement and control of corneal thickness.. Invest Ophthalmol Vis Sci.

[32] Wigham CG, Turner HC, Swan J, Hodson SA (2000). Modulation of corneal endothelial hydration control mechanisms by Rolipram.. Pflugers Arch.

[33] Blachly-Dyson E, Forte M (2001). VDAC channels.. IUBMB Life.

[34] Gonzalez-Gronow M, Kalfa T, Johnson CE, Gawdi G, Pizzo SV (2003). The voltage-dependent anion channel is a receptor for plasminogen kringle 5 on human endothelial cells.. J Biol Chem.

[35] Thiagarajah JR, Verkman AS (2002). Aquaporin deletion in mice reduces corneal water permeability and delays restoration of transparency after swelling.. J Biol Chem.

[36] Wigham CG, Guggenheim JA, Hodson SA (1994). Sodium movement into and out of corneal endothelium.. Pflugers Arch.

[37] Montcourrier P, Hirsch M (1985). Intercellular junctions in the developing rat corneal endothelium.. Ophthalmic Res.

[38] Petroll WM, Hsu JK, Bean J, Cavanagh HD, Jester JV (1999). The spatial organization of apical junctional complex-associated proteins in feline and human corneal endothelium.. Curr Eye Res.

[39] Jongen WM, Fitzgerald DJ, Asamoto M, Piccoli C, Slaga TJ, Gros D, Takeichi M, Yamasaki H (1991). Regulation of connexin-43-mediated gap junctional intercellular communication by Ca^2+^ in mouse epidermal cells is controlled by E-cadherin.. J Cell Biol.

[40] Rayner SA, Gallop JL, George AJT, Larkin DFP (1998). Distribution of integrins alpha v beta 5, alpha v beta 3 and alpha v in normal human cornea: possible implications in clinical and therapeutic adenoviral infection.. Eye.

[41] Chen WL, Lin CT, Lo HF, Lee JW, Tu IH, Hu FR (2007). The role of protein tyrosine phosphorylation in the cell-cell interactions, junctional permeability and cell cycle control in post-confluent bovine corneal endothelial cells.. Exp Eye Res.

[42] Zhu YT, Hayashida Y, Kheirkhah A, He H, Chen SY, Tseng SC (2008). Characterization and comparison of intercellular adherent junctions expressed by human corneal endothelial cells *in vivo* and *in vitro.*. Invest Ophthalmol Vis Sci.

[43] Vrana NE, Builles N, Justin V, Bednarz J, Pellegrini G, Ferrari B, Damour O, Hulmes DJ, Hasirci V (2008). Development of a reconstructed cornea from collagen-chondroitin sulfate foams and human cell cultures.. Invest Ophthalmol Vis Sci.

